# Targeting NETs using dual-active DNase1 variants

**DOI:** 10.3389/fimmu.2023.1181761

**Published:** 2023-05-23

**Authors:** Hanna Englert, Josephine Göbel, Danika Khong, Maryam Omidi, Nina Wolska, Sandra Konrath, Maike Frye, Reiner K. Mailer, Manu Beerens, Julian C. Gerwers, Roger J. S. Preston, Jacob Odeberg, Lynn M. Butler, Coen Maas, Evi X. Stavrou, Tobias A. Fuchs, Thomas Renné

**Affiliations:** ^1^ Institute of Clinical Chemistry and Laboratory Medicine, University Medical Center Hamburg-Eppendorf, Hamburg, Germany; ^2^ Irish Centre for Vascular Biology, School of Pharmacy and Biomolecular Sciences, Royal College of Surgeons in Ireland, Dublin, Ireland; ^3^ Department of Clinical Medicine, The Arctic University of Norway, Tromsø, Norway; ^4^ Science for Life Laboratory, Department of Protein Science, CBH, KTH Royal Institute of Technology, Stockholm, Sweden; ^5^ Department of Molecular Medicine and Surgery, Karolinska Institute, Stockholm, Sweden; ^6^ Department of Clinical Chemistry and Haematology, University Medical Center Utrecht, Utrecht University, Utrecht, Netherlands; ^7^ Medicine Service, Section of Hematology-Oncology, Louis Stokes Veterans Administration Medical Center, Cleveland, OH, United States; ^8^ Department of Medicine, Hematology and Oncology Division, Case Western Reserve University School of Medicine, Cleveland, OH, United States; ^9^ Neutrolis, Inc., Cambridge, MA, United States; ^10^ Center for Thrombosis and Hemostasis (CTH), Johannes Gutenberg University Medical Center, Mainz, Germany

**Keywords:** neutrophil extracellular traps (NETs), NETosis, NET degradation, DNase1, DNase1-like 3, thromboinflammation, protein engineering, recombinant proteins

## Abstract

**Background:**

Neutrophil Extracellular Traps (NETs) are key mediators of immunothrombotic mechanisms and defective clearance of NETs from the circulation underlies an array of thrombotic, inflammatory, infectious, and autoimmune diseases. Efficient NET degradation depends on the combined activity of two distinct DNases, DNase1 and DNase1-like 3 (DNase1L3) that preferentially digest double-stranded DNA (dsDNA) and chromatin, respectively.

**Methods:**

Here, we engineered a dual-active DNase with combined DNase1 and DNase1L3 activities and characterized the enzyme for its NET degrading potential in vitro. Furthermore, we produced a mouse model with transgenic expression of the dual-active DNase and analyzed body fluids of these animals for DNase1 and DNase 1L3 activities. We systematically substituted 20 amino acid stretches in DNase1 that were not conserved among DNase1 and DNase1L3 with homologous DNase1L3 sequences.

**Results:**

We found that the ability of DNase1L3 to degrade chromatin is embedded into three discrete areas of the enzyme's core body, not the C-terminal domain as suggested by the state-of-the-art. Further, combined transfer of the aforementioned areas of DNase1L3 to DNase1 generated a dual-active DNase1 enzyme with additional chromatin degrading activity. The dual-active DNase1 mutant was superior to native DNase1 and DNase1L3 in degrading dsDNA and chromatin, respectively. Transgenic expression of the dual-active DNase1 mutant in hepatocytes of mice lacking endogenous DNases revealed that the engineered enzyme was stable in the circulation, released into serum and filtered to the bile but not into the urine.

**Conclusion:**

Therefore, the dual-active DNase1 mutant is a promising tool for neutralization of DNA and NETs with potential therapeutic applications for interference with thromboinflammatory disease states.

## 1 Introduction

A delicate network of mechanisms that mediate both thrombosis and inflammation underlies diseases such as stroke, deep vein thrombosis, atherosclerosis and myocardial infarction, sepsis, disseminated intravascular coagulation (DIC), and cancer associated thrombosis. This intimate crosstalk of procoagulant and proinflammatory responses has been termed thromboinflammation (1–3). As a result, genetic or pharmacologic interference with thromboinflammatory pathways has emerged as a promising new strategy for the prevention and treatment of an array of disease states characterized by exuberant procoagulant and proinflammatory responses (4, 5). There is a diverse repertoire of humoral and cell-derived mediators driving inflammation *in vivo*, among them neutrophil extracellular traps (NETs) (6). To battle infection and inflammation, neutrophils release NETs, supramolecular extracellular lattices of DNA filaments complexed with histones and neutrophil granule proteins such as myeloperoxidase and neutrophil elastase (7). NET formation can be triggered by a variety of stimuli *in vivo*, including whole bacteria and fungi, bacterial cell surface components such as lipoteichoic acid and lipopolysaccharide (LPS), breakdown products of prokaryotic proteins such as N-formyl-methionyl-phenylalanine (fMLP), immune complexes, or interleukins and chemokines such as interleukin-8 (IL-8) or tumor necrosis factor alpha (TNFα) (8). Beyond their importance for the resolution of infection, NETs have been implicated in the pathology of an array of (auto)immune diseases and have been shown to contribute to a growing number of infectious disease states (9–11). DNA fibers in NETs initiate thrombo-occlusive mechanisms with critical implications for organ perfusion in septic conditions (12).

Removal of NETs from the circulation is crucial to prevent the host from hyperinflammation, immunothrombosis, and auto-immune reactions (13–15). Due to their potentially detrimental activities, NET turnover is tightly controlled by circulating extracellular DNases that degrade NETs (16). The critical role of DNases for neutralizing NETs is underlined by their presence in pathogenic bacteria, that secrete NET-cleaving DNases to facilitate their escape from the host defense system (17). The enzymes responsible for clearing antigenic nucleic acids and NETs from circulation are the DNase1 protein family members DNase1 and DNase1L3 (also called DNase1-like 3, DNase γ, LS-DNase). Mice lacking both DNase1 and DNase1L3, but not single gene-deficient or wild type (WT) animals, are susceptible to NET-mediated vascular occlusions in models of lethal thromboinflammation (18). Recombinant human DNase1 (dornase alfa, Pulmozyme, Roche) is marketed for the treatment of cystic fibrosis (CF) patients. The mucolytic activity of Pulmozyme is however inhibited by actin in the sputum of CF patients, suggesting that an actin-resistant DNase variant would offer an alternative and potentially more effective therapeutic option (19).

Enzymes of the DNase1 family produce DNA cleavage products with 5’-P and 3’-OH ends (20) with the primary substrate being the canonical right-handed B-DNA, > 10 nucleotides long stretches with a tendency towards non-repeating base pairs (21). However, DNase1 family members differ in their substrate preference as studied for DNase1 and DNase1L3 (16). The catalytic activity of DNase1 is mainly directed toward double-stranded DNA (dsDNA), such as plasmid or bacterial DNA and the dsDNA linking chromatin aggregates within NETs. In contrast, DNase1L3 is less selective and digests a range of substrates including naked dsDNA and complexed DNA, i.e., bound to proteins in chromatin and nucleosomes, lipid-coated DNA and DNA components of microparticles or apoptotic bodies (22–24). Besides their specific substrate affinities, DNase1 and DNase1L3 have unique structural properties that determine their distinct enzymatic functions. It is believed that the positively charged C-terminal domain (CTD) of DNase1L3, containing two nuclear localization signals (NLS1 and NLS2) and a rigid α-helix, allows for degradation of DNA complexed with proteins and lipids more efficiently than DNase1 (23–25). In contrast, DNase1 is devoid of a C-terminal helix or NLS, and therefore defective in binding to complexed DNA in microparticles or the nucleus (23). DNase1 is inhibited by actin, while DNase1L3 is inactivated by heparin and can be cleaved by plasmin (22).

DNase1 preferentially digests naked dsDNA, whereas chromatin is the substrate for DNase1L3. Efficient NET degradation requires the combined action of both enzymes. To exploit the functional differences between DNase1 and DNase1L3, we aimed to generate recombinant human DNase1/DNase1L3 variants that combine key properties of both enzymes and offer a therapeutic advantage over their combined use. We systematically substituted single or multiple non-conserved amino acids in DNase1 with the corresponding sequence of DNase1L3. Our primary readouts “single radial enzymatic diffusion” (SRED) zymography and chromatin degradation assays were employed to screen for DNase1 and DNase1L3 activities in these mutants. We identified three non-conserved amino acid stretches that were sufficient in transferring the substrate specificity of DNase1L3 to DNase1. We produced mice with transgene expression of a DNase1 mutant carrying a combination of all three DNase1L3 amino acid stretches without endogenous DNase activity, and detected activity in serum and bile but not in urine. The DNase1 mutant potently degraded NETs more efficiently than both DNase1 and DNase1L3, suggesting therapeutic potential of the novel DNase1 variant for interference with NET-mediated disease states.

## 2 Results

### 2.1 Design of dual-active DNase1 variants

**Figure 1:**
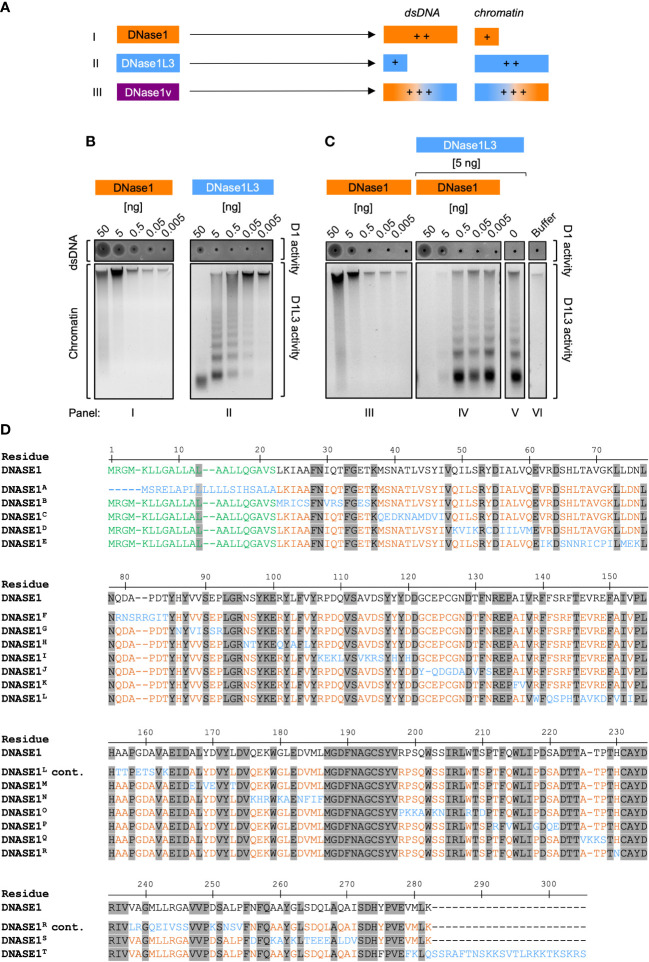
Strategy for engineering dual-active DNase1/DNase1L3 mutants. **(A)** Scheme for the generation of dual-active DNase mutants based on addition of DNase1L3 activity to DNase1. DNase1 (I, orange) and DNase1L3 (II, blue) differ in their substrate specificities: DNase1 degrades dsDNA more efficiently than chromatin, and *vice versa* chromatin DNA is a preferred substrate for DNase1L3 as compared to dsDNA. The designed dual-active DNase1 variant (DNase1v, III, purple) contains sequences of DNase1 and DNase1L3 and efficiently digests both dsDNA, and chromatin. **(B)** Purified recombinant DNase1 (*panel I)* and DNase1L3 (*panel II*; 50 - 0.005 ng) were either incubated with dsDNA or chromatin isolated from salmon testes or from HEK293 cell nuclei, respectively. Diameters of the dark circles in zymography SRED assays present dsDNA degrading DNase1 activity (upper gel; dsDNA/D1activity). Agarose gel electrophoresis of digested chromatin shows chromatin degrading activity of DNase1L3. High-molecular weight chromatin complexes are cleaved into smaller fragments (lower gel; chromatin/D1L3 activity). **(C)** Co-incubation of DNase1L3 and DNase1 shows synergistic DNases activities. dsDNA and chromatin were incubated with increasing levels of DNase1 alone (*panel III*) or together with DNase1L3 (5 ng, *panel IV*) that alone (*panel V*) was not sufficient to degrade chromatin completely. *Panel VI* shows buffer treated dsDNA and chromatin. **(D)** Amino acid sequences of DNase1 and mutated regions of the 20 engineered DNase1 variants (DNase1^A^ – DNase1^T^, which are detailed in **Supplementary Table 1**). Numbering relates to native DNase1 sequence. Residues conserved between DNase1 and DNase1L3 are depicted in grey and the signal sequence of DNase1 is highlighted in green. Non-conserved residues are highlighted in orange in the DNase1 backbone. DNase1L3 sequence stretches that were swapped into the DNase1 backbone are shown in blue. “cont.” indicates that DNase1^L^ and DNase1^R^ sequences continue from the lines above. D1 – DNase1, D1L3 – DNase1L3.

We aimed to generate a DNase molecule that combines DNase1L3 and DNase1 substrate specificities to improve NET degradation capacity. Transfer of DNase1L3 specific residues into a DNase1 backbone was predicted to result in a DNase variant that effectively degrades both dsDNA and chromatin resulting in rapid clearance of NETs (**Figure 1A**). To confirm substrate specificity of DNase1 and DNase1L3 and test for potential synergistic activity, we analyzed recombinantly produced proteins using as readouts (i) dsDNA degradation with SRED zymography and (ii) cleavage of chromatin, respectively.

As expected, recombinant DNase1 dose-dependently cleaved dsDNA (**Figure 1B**, I; upper gel). The circle diameters in SRED zymography were measured by densitometric scans and are positively correlated with DNase1 activity. In samples with minor activity, the loading well appeared as a black dot without a halo (**Figure 1B**, I and II; upper gels). DNase1 was largely inactive in degrading chromatin and the highest concentration of the enzyme (50 ng) slightly digested loaded chromatin (**Figure 1B**, I, lower gel, first lane from the left). Digestion of chromatin by DNase1 also led to an increase in fluorescence of the DNA-intercalating SYBR Safe dye (**Figure 1B**, I; lower gel; lanes 50 and 5 ng). Purified DNase1L3 (50 ng) completely degraded chromatin into small fragments, concomitant by the disappearance of the high molecular weight DNA aggregate and the emergence of a typical DNA ladder pattern, but had minimal dsDNA degrading activity (**Figure 1B**, II; upper and lower gel). Concluding from the zymographic and fluorescence data, DNase1 degrades dsDNA approximately 100-fold more efficiently as compared to DNase1L3, while the latter is approximately 100-fold more potent over DNase1 in degrading chromatin. We compared DNase1-mediated chromatin degradation in the absence (**Figure 1B**, III; lower gel) or presence (**Figure 1B**, IV; lower gel) of DNase1L3. Importantly, when low amounts of DNase1L3 (5 ng; not sufficient for degrading chromatin completely, **Figure 1C**, V) were co-incubated with DNase1, chromatin was completely digested (≥5 ng purified DNase1 protein; **Figure 1C**, IV; lane 1 and 2), indicating synergistic activities for DNase1 and DNase1L3.

Residue alignment of DNase1 and DNase1L3 amino acid sequences revealed 44% homology between the enzymes (**Supplementary Figure 1**). We systematically exchanged stretches of non-conserved sites in DNase1 with amino acid residues of DNase1L3 but did not alter sequences that were conserved among both DNases. Sequences of the resulting 20 DNase1 mutants harboring the substituted DNase1L3 residues are shown in **Figure 1D**. **Supplementary Table 1** gives a more detailed summary of the exchanged and conserved residues in all DNase1/DNase1L3 variants that we produced by *de novo* synthesis, e.g., in DNase1^H^ variant sites 15 (N^96^S^97^), 16 (R^101^), 17 (L^103^), and 18 (V^105^) in DNase1 were substituted with corresponding residues 15´ (N^96^T^97^), 16´ (Q^101^), 17´ (A^103^), and 18´ (L^105^) of DNase1L3.

### 2.2 Screening of dual-active DNase1 variants

**Figure 2:**
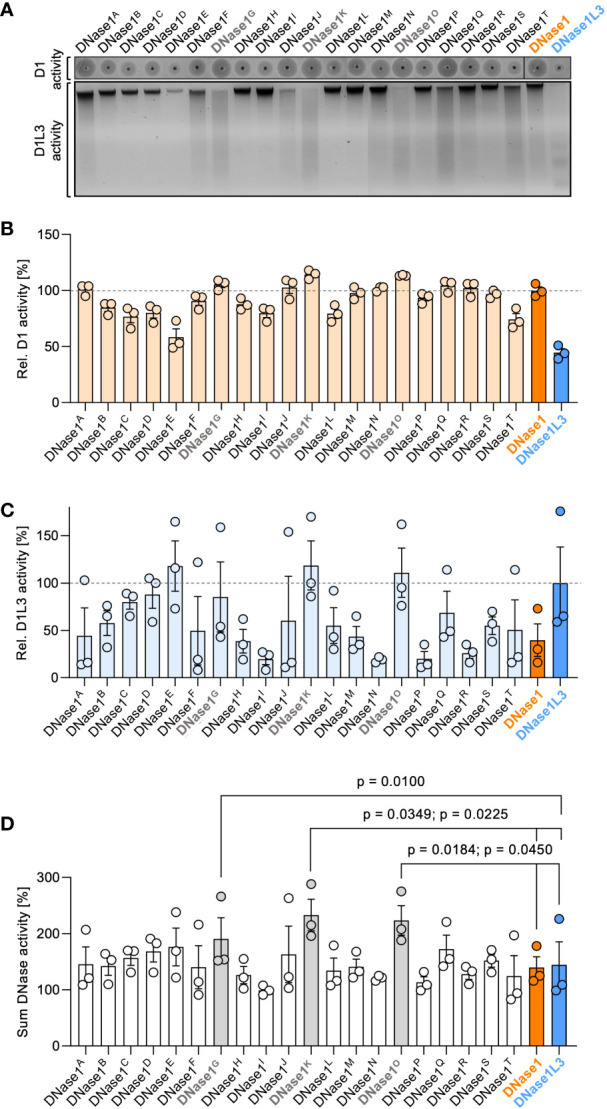
Screening of dual-active DNase1 variants for DNase1 and DNase1L3 activity. **(A)** dsDNA (upper gel; D1 activity) or chromatin (lower gel; D1L3 activity) was incubated with supernatants of HEK293 cell-expressed DNase1 variants, DNase1 or DNase1L3. DNase1 and DNase1L3 activities of the various DNases were analyzed as in **Figure 1B** above. **(B)** Densitometric scans of the digested dsDNA quantified DNase1 activity. Enzymatic activity is given relative to DNase1 (D1) levels set to 100%. **(C)** Residual chromatin signals as a measure for DNase1L3 activity is blotted relative to DNase1L3 (D1L3) activity (100%). **(D)** Sum of DNase1 and DNase1L3 activities derived from **(B)** and **(C)**. *p*-value by paired one-way ANOVA with Dunnett’s multiple comparisons test. Data are mean ± SEM, n = 3. D1 – DNase1, D1L3 – DNase1L3.

We recombinantly expressed the designed DNase1 variants in human embryonic kidney (HEK) 293 cells and systematically screened the cell supernatants for their DNase1 and DNase1L3 activity using SRED zymography (**Figure 2A**, upper gel) and chromatin degradation (**Figure 2A**, lower gel), respectively. DNase1 enzymatic activities of DNase1^B^, DNase1^D^, DNase1^E^, DNase1^I^, DNase1^L^, and DNase1^T^ were decreased, whereas DNase1^K^ and DNase1^O^ showed a significantly higher enzymatic activity compared to native DNase1. DNase1L3 activity of variants DNase1^H^, DNase1^I^, DNase1^M^, DNase1^N^, DNase1^P^, DNase1^R^, and DNase1^S^ were lower compared to native DNase1L3 and none of the variants significantly exceeded the enzymatic activity of native DNase1L3 (**Supplementary Table 2**). DNase1 activities of variants DNase1^A^ (101%), DNase1^G^ (105%), DNase1^J^ (103%), DNase1^K^ (114%), DNase1^N^ (102%), DNase1^O^ (113%), DNase1^Q^ (104%) and DNase1^R^ (102%) exceeded non-mutated, native DNase1 levels (set to 100%; **Figure 2B**). Next, the variants were analyzed for their DNase1L3 activity. Chromatin-digesting activities of DNase1^E^ (118%), DNase1^K^ (119%), and DNase1^O^ (111%) exceeded levels of non-mutated, native DNase1L3 (100%; **Figure 2C**). As some mutations conferred opposite effects on DNase1 and DNase1L3 activities, variants with the highest cumulative DNase1 and DNase1L3 activity were selected for subsequent studies. Among these, combined DNase1 and DNase1L3 activities of mutants DNase1^G^ (191%), DNase1^K^ (233%), and DNase1^O^ (224%) were significantly increased compared to total levels of DNases (**Figure 2D**).

### 2.3 Characterization of the dual-active variant DNase1^G,K,O^

**Figure 3:**
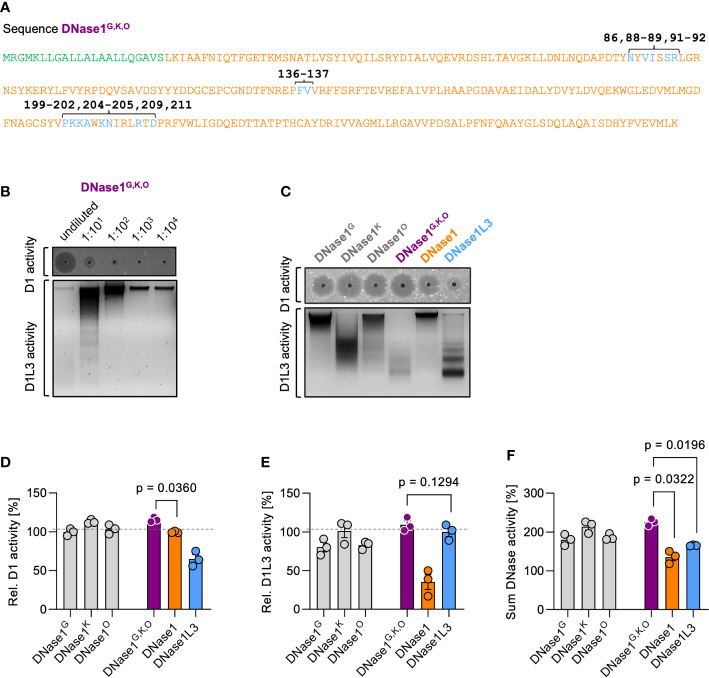
Characterization of dual-active variant DNase1^G,K,O^. **(A)** Amino acid sequence of the dual-active DNase1 variant DNase1^G,K,O^ that combines the identified DNase1L3-derived mutations of DNase1^G^ (five residues; positions 86, 88-89, 91-92), DNase1^K^ (two residues; positions 136-137) and DNase1^O^ (eight residues; positions 199-202, 204-205, 209, 211). Signal peptide sequence is shown in green. Non-mutated DNase1 sequence is in orange and swapped DNase1L3 derived residues in blue. **(B)** Dose dependency of dsDNA (upper gel; D1 activity) and chromatin (lower gel; D1L3 activity) degrading activity of DNase1^G,K,O^. Serial 1:10 dilution of supernatants of transfected HEK293 cells were tested. **(C)** dsDNA (upper gel; D1 activity) and chromatin (lower gel; D1L3 activity) degrading activity of recombinant DNase1^G^, DNase1^K^, DNase1^O^, and DNase1^G,K,O^ variants, and native DNase1, and DNase1L3. **(D)** Quantification of DNase1, **(E)** DNase1L3, and **(F)** combined DNases activities of various DNases from densitometric scans as in **(C)**. Data is given relative to native enzyme activity (100%) in **(D)** and **(E)**. *p*-value by paired one-way ANOVA with Dunnett’s multiple comparisons test. Columns represent mean ± SEM, n = 3. D1 – DNase1, D1L3 – DNase1L3.

We next analyzed the combined effects of the three mutated sequences. Substitutions of DNase1 with the corresponding DNase1L3 amino acid stretches G (H^86^ by N^86^; V^88^V^89^ by V^88^I^89^ and E^91^P^92^ by S^91^R^92^), K (A^136^I^137^ by F^135^V^136^), O (R^199^P^200^S^201^Q^202^ by P^198^K^199^K^200^A^201^; S^204^S^205^ by K^203^N^204^ and W^209^ by R^208^; S^211^ by D^210^) comprise a total of 15 amino acid residues, which were combined into one novel variant DNase1^G,K,O^ (**Figure 3A**; DNase1L3 sequence is in blue and the original DNase1 backbone in orange). SRED zymography and chromatin degradation showed that DNase1^G,K,O^ potently degraded both dsDNA and chromatin, in a dose-dependent manner (**Figure 3B**). DNase1 activity of variant DNase1^G,K,O^ significantly exceeded that of the native enzyme (116 *vs.* 100%, *p* = 0.0360) and showed a trend towards superior degrading potential compared to DNase1^G^, DNase1^K^ and DNase1^O^ mutants that each harbor mutations in an individual amino acid stretch (**Figure 3C** upper gel and **Figure 3D**; 100%, 113% and 103%). Despite having only 15 residues of the original DNase1L3 sequence, the DNase1^G,K,O^ mutant showed a trend towards higher DNase1L3 activity compared to the native enzyme and mutants with single variations (**Figure 3C** lower gel and **Figure 3E**; 110 *vs.* 100%, 80%, 101% and 83%). Notably, combined DNase1 and DNase1L3 activities of DNase1^G,K,O^ exceeded levels of DNase1 and DNase1L3 (**Figure 3F**; 225 *vs.* 135% and 165%, *p* = 0.0322 and *p* = 0.0196). We next compared our dual-active DNase1 variant to DNase1 and DNase1L3 *in vivo*.

### 2.4 Dual-active DNase1^G,K,O^ variant is stable and functional in serum and bile but not in urine of transgenic mice

**Figure 4:**
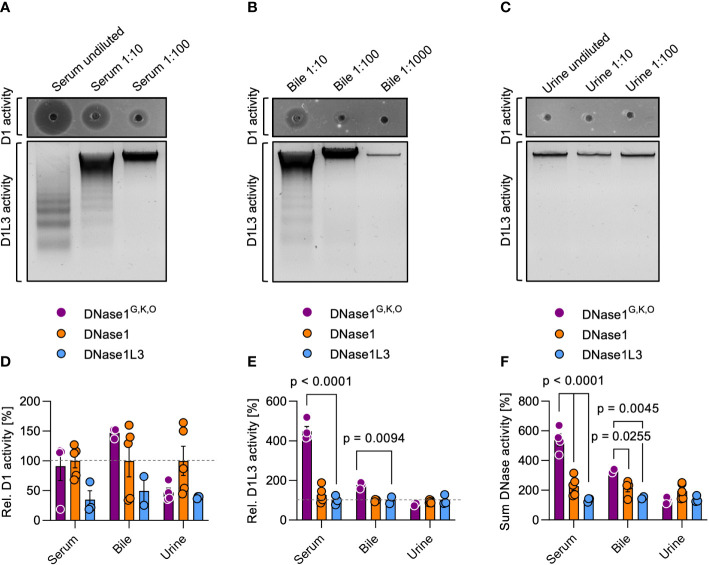
DNase activities in serum, bile, and urine of DNase1^G,K,O^ transgenic mice. We produced mouse lines with transgenic expression of DNase1^G,K,O^, DNase1 and DNase1L3 in hepatocytes on a *Dnase1^-/-^Dnase1l3^-/-^* deficient background. DNase1 (upper gel; D1 activity) and DNase1L3 (lower gel; D1L3 activity) activities in serum **(A)**, bile **(B)**, and urine **(C)** of DNase1^G,K,O^ transgenic mice were measured by dsDNA and chromatin degradation, respectively. Representative gels of n = 3. DNase1 **(D)**, DNase1L3 **(E)** and summarized DNase1 and DNase1L3 **(F)** activities in serum, bile, and urine of DNase1^G,K,O^ (purple) DNase1 (orange) and DNase1L3 (blue) transgenic mice. Activities are normalized to native DNase levels (100%). Data represent mean ± SEM, *p*-value by two-way ANOVA with Dunnett’s multiple comparisons test. Each data point represents an individual animal, DNase1^G,K,O^ (n = 4), DNase1 (n = 5), DNase1L3 (n = 3). D1 – DNase1, D1L3 – DNase1L3.

To test whether DNase1^G,K,O^ can serve as a tool to target NETs *in vivo*, we transgenically expressed DNase1^G,K,O^ in hepatocytes of *Dnase1^-/-^Dnase1l3^-/-^* mice that are deficient in endogenous DNases (18). We collected serum, bile and urine of the three transgenic mouse lines and analyzed the biological fluids for dsDNA and chromatin degradation activity. DNase activity was not detectable in *Dnase1^-/-^Dnase1l3^-/-^* mice (18). In contrast, sera from DNase1^G,K,O^ transgenic mice dose-dependently degraded both dsDNA and chromatin (**Figure 4A**). Similarly, DNase1 and DNase1L3 activities were detected in bile, albeit at lower levels, but not in the urine of DNase1^G,K,O^ transgenic mice (**Figures 4B, C** **)**. DNase1 activity of DNase1^G,K,O^ expressing mice was similar in serum and slightly higher in bile than in DNase1 transgenic animals (100%, **Figure 4D**), while a significant increase in DNase1L3 activity was found in serum and bile of animals expressing DNase1^G,K,O^ compared to DNase1L3 transgenic mice (100%, **Figure 4E** **)**. DNase1 activity in serum of DNase1^G,K,O^ transgenic mice exceeded levels seen in WT mice by ~100-fold, while DNase1L3 activity was largely elevated in WT mouse serum compared to DNase1^G,K,O^ transgenic mice (data not shown). Notably, combined DNase1 and DNase1L3 activities surpassed levels of each native enzyme in sera and bile of DNase1^G,K,O^ expressing animals (**Figure 4F**; 540 *vs.* 228% and 135% in serum, *p* < 0.0001 each; 329 *vs.* 216% and 150% in bile, *p* = 0.0255, *p* = 0.0045, respectively). These *in vivo* data demonstrate that engineered DNase1^G,K,O^ is secreted into body fluids, and that the enzyme is stable and functional in serum and bile.

### 2.5 DNase1^G,K,O^ is superior to DNase1 and DNase1L3 in degrading human NETs

**Figure 5:**
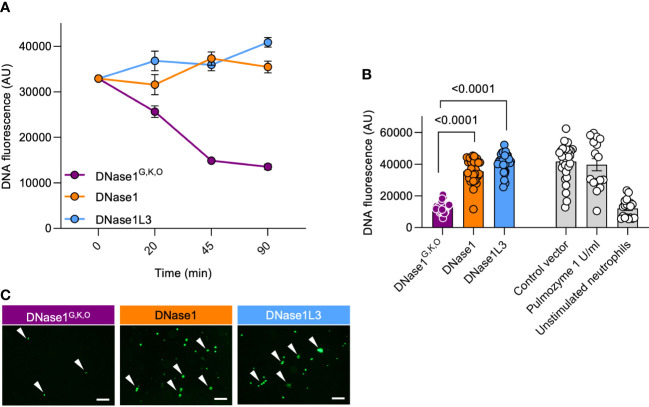
Dual-active DNase1^G,K,O^ efficiently degrades NETs. Human neutrophils were PMA-stimulated to produce NETs. Formed NETs were treated with supernatants of DNase1^G,K,O^, DNase1, DNase1L3, or of mock vector transfected HEK293 cells. **(A)** Kinetics of DNase1^G,K,O^, DNase1 and DNase1L3-mediated NET degradation. Data represent mean ± SEM. **(B)** Residual NET fibers upon DNase1^G,K,O^, DNase1, DNase1L3 incubation (n = 32, each) were quantified by Sytox Green fluorescence at 90 min. Recombinant human DNase1 (1 U/ml, Pulmozyme; n = 16) and baseline fluorescence of unstimulated neutrophils (n = 20) served as controls. Data represent mean ± SEM, *p*-value, Kruskal-Wallis with Dunn’s multiple comparisons test. **(C)** Representative fluorescence images of NETs after 90 min DNase treatment. White arrowheads indicate undigested NET remnants. A representative image of n = 3 is shown. Scale bar 100 µm.

We next aimed to characterize the capacity of DNase1^G,K,O^ for degrading NETs. Healthy human neutrophils were isolated, activated with 100 nM PMA for 4 hours to induce NETosis (26) and incubated with HEK293 cell-produced DNase1, DNase1L3, or DNase1^G,K,O^. Supernatants of mock vector transfected cells served as negative controls. Recombinant DNase1^G,K,O^ but not DNase1 and DNase1L3 incubation time-dependently degraded NETs (13,522 mean fluorescence in arbitrary units (AU) *vs.* 35,465 AU and 40,882 AU at 90 min; *p* < 0.0001 each, **Figure 5A**). NET signal was lowest following 90 min incubation, and this condition was used for all further experiments. Consistently, DNase1^G,K,O^ degraded formed NETs as seen by changes in fluorescence of intact DNA fibers while DNase1 and DNase1L3 cell supernatants were inactive (**Figure 5B**). Activity of DNase1^G,K,O^ abolished DNA fluorescence signal to levels of unstimulated neutrophils (12,078 AU), while DNase1 and DNase1L3 activities were close to levels of mock vector transfected cell supernatants (41,615 AU). Commercially available DNase1 (1 U/ml, Pulmozyme) was not superior compared to DNase1^G,K,O^ in degrading NETs (39,664 AU). Representative nucleic acid staining confirmed that the potency of DNase1^G,K,O^ in degrading NETs exceeded that of DNase1 and DNase1L3 (**Figure 5C**).

## 3 Discussion

Abundant NETs are found in thrombi of patients with ischemic stroke (27, 28) and myocardial infarction (29). NETs provide a scaffold for platelet and erythrocyte adhesion, locally enrich coagulation factors and confer resistance to fibrinolysis (28, 30–34). Treatment with recombinant human DNase1 allowed for the recanalization of occluded vessels and improved prognosis in murine stroke models (35, 36), motivating therapeutic use of DNase mutants for thromboprotection. We identified three sites in DNase1L3 that allow for transfer of chromatin-degrading activity to DNase1. *De novo* synthesis led to the stable, plasma-borne, dual-active DNase1 chimera DNase1^G,K,O^ that efficiently cleaved both dsDNA and chromatin, and potently degraded NETs. Our study established a novel and promising *in vivo* tool for interference with NET-associated diseases in the arterial and venous circulation and bile ducts.

Our engineered variant DNase1^G,K,O^ comprises the three mutant building blocks G, K, and O (**Supplementary Table 1**, **Figure 2**). Sequences G and K include amino acid residues H86 - R95 and A136 - V138 that mediate binding of DNase1 to its inhibitor G-actin. In contrast to DNase1, DNase1L3 does not interact with actin (37). Actin fibers and DNA share filament helicity and histones bundle both, actin and DNA supporting similarities in structure among the polyanionic fibers (38–40). Thus, exchange of DNase1 regions by sequences lacking actin binding regions, increases its activity, because the enzyme is less prone to inhibition by actin. In 1996, researchers from Genentech (San Francisco, CA, USA) engineered actin-resistant DNase1 variants which reduced the viscoelasticity of CF patient sputum 10- to 50-fold more potently than native DNase1 (41). Actin-resistant DNase1 variants also have a therapeutic benefit in lupus nephritis models (42, 43). The sequence stretch O (P*KK*AW*K*NIRLRTD, **Supplementary Table 1**) that we transferred from DNase1L3 to DNase1 (substituting RPSQWSSIRLWTS) entails three lysine residues absent in the original DNase1 (44) sequence. Addition of three positively charged amino acids to DNase1 increases binding affinity of mutant DNase1^O^ for its substrate DNA resulting in a gain of DNA-degrading activity. Challenging the current dogma that the C-terminal domain (CTD) mediates DNase1L3 activity (23, 24), our mutants identified transferrable regions in the core body of the enzyme that comprise chromatin cleavage activity (**Figure 3**) with possible *in vivo* relevance (**Figure 4**). The mechanism on how these amino acid substitutions confer DNase1L3 activity needs to be further investigated using protein structure analyses. In contrast to DNase variants with novel, *de novo* designed sequences, our strategy relies on combinations of existing protein sequences (**Figure 1**). Combining naturally existing sequences results in DNase variants that likely have less immunogenic activity to trigger autoantibody production. Preliminary data reveal that transgenically expressed DNase1^G,K,O^ is detectable in serum and bile one week post hydrodynamic tail vein injection suggesting that the engineered construct induces little if any autoantibody production (**Figure 4**). The *in vivo* data support further pharmacological studies including long term treatment and repeated application. In addition to a prolonged half-life, strategies for targeting recombinant DNases to specific sites of inflammation will improve potential clinical use of the DNase protein family. Indeed, a recombinant bovine pancreatic DNase1 fused to single-chain Fv antibody fragments directed against human placental alkaline phosphatase is stable and the chimeric molecule displayed both DNA-degrading and antigen-binding activity *in vitro*. In cell culture the DNase1-fusion protein exerts high cytotoxicity towards cells expressing placental alkaline phosphatase (45).

Defective DNase activity prolongs the half-life of circulating cell-free DNA, NETs and anti-dsDNA antibodies that all contribute to autoimmune disease mechanisms (8). Indeed, deficiency in members of the DNase1 family is a risk factor for hypocomplementemic urticarial vasculitis syndrome (HUVS), hemolytic uremic syndrome (HUS), lupus nephritis and systemic lupus erythematosus (SLE) (13, 15, 46, 47). Congenital DNase1L3 deficiency predisposes individuals to pediatric-onset SLE (48). Similarly, combined deficiency in DNase1 and DNase1L3 due to neutralizing auto-antibodies, impairs NET clearance and underlies the inflammatory skin disorder hidradenitis suppurativa (acne inversa, 49). Consistently, polymorphisms in DNase1L3 and DNase1 genes predispose to the development of dsDNA/NET-mediated adult-onset SLE (47, 50, 51). Supporting a role of DNases in autoimmunity, deficiency in DNase1 or DNase1L3 triggers lupus-like symptoms in ANA-positive glomerulonephritis and promotes kidney deposition of immune complexes and complement factor C3 in genetically modified mice (14). Combined deficiency in the B lymphocyte inhibitory receptor Siglec-G and DNase1L3 was shown to accelerate manifestations of SLE and increased anti-dsDNA antibody and ANA-titers, suggesting cooperative effects among these genes in murine models (52).

Both human and murine data suggest that DNases can have clinical utility in autoimmune disease states. Indeed, the U.S. Food and Drug Administration (FDA) has approved therapeutic use of a recombinant human DNase1 (dornase alfa, Pulmozyme). Inhaled Pulmozyme digests DNA in the alveolar space, reduces mucus viscosity and improves gas exchange and oxygenation in cystic fibrosis (CF) patients (53–55). Off-label use of Pulmozyme has also previously shown efficacy in patients suffering from NET-associated respiratory diseases such as asthma, atelectasis and COVID-19 pneumonia (56–59). Previously, we showed that DNase deficiency led to defective NET clearance and augmented thromboinflammation in COVID-19 patient lungs (60). *Vice versa*, inhaled Pulmozyme improved patient oxygenation but also increased virus spreading in COVID-19 pneumonia (61). Similarly, intrapleural administration of Pulmozyme in empyema patients reduced pus viscosity and facilitated drainage of effusions, suggesting a role of NETs in the disease (62).

While Pulmozyme therapy is beneficial in pulmonary diseases, a phase Ib clinical trial in lupus nephritis patients found no preliminary benefit in the Pulmozyme-treated arm over placebo. Specifically, while both subcutaneous and intravenous DNase1 application proved safe, disease biomarkers including dsDNA antibodies, complement factors C3 and C4, and various cytokines were not significantly different among groups over the prespecified 40-day time period (63). There are ongoing clinical trials evaluating Pulmozyme, among them studies to assess its effect on cerebral perfusion in stroke patients following endovascular thrombectomy (NCT04785066; NCT05203224), based on the finding that Pulmozyme improved hindlimb perfusion in experimental ischemia/reperfusion mouse models (64).

It is important to note that our study does not evaluate the DNase1^G,K,O^ variant *in vivo*, in experimental thromboinflammation models. However, we envision that the newly developed DNase1^G,K,O^ could be therapeutically beneficial in NET-associated thromboinflammatory disorders such as ischemic stroke, deep vein thrombosis and myocardial infarction. The occurrence of DNase1^G,K,O^ variant in bile raises the possibility that sufficient levels can be achieved to target NETs in inflammatory biliary diseases such as primary sclerosing cholangitis. In future studies, we plan to analyze the therapeutic potential of DNase1^G,K,O^ in thromboinflammation and sepsis models *in vivo*. In summary, we have designed a recombinant human DNase1 variant that efficiently degrades both dsDNA and chromatin, with potential therapeutic applications for interference with cell-free DNA-, and NET-driven disease states.

## 4 Materials and methods

### 4.1 Detection of DNase1 activity by single radial enzyme diffusion assay

To measure DNase1 activity, we dissolved 0.13 mg/ml DNA from salmon testes (D1626, Sigma) in a buffer containing Mg^2+^ (20 mM Tris-HCl pH 7.8, 10 mM MgCl_2_, 2 mM CaCl_2_, and 2 x SYBR Safe). The DNA solution was heated to 50°C for 10 min and mixed with an equal volume of 2% ultrapure agarose (16500500, Thermo Scientific). The mixture was cast into plastic trays and covered with a silicon mat with small protrusions to generate 1.0 mm diameter wells in the gel. The gel was stored at room temperature until solidification. Two microliters of HEK293 cell supernatant or purified DNases were loaded into wells. DNA was incubated for 3 h at 37°C in a humid chamber and fluorescence was recorded with the Bio-Rad ChemiDoc Imager. ImageJ was used for the quantification of the diameter of the circles reflecting DNase activity.

### 4.2 Detection of DNase1L3 activity by chromatin degradation assay

To assess DNase1L3 activity we isolated nuclei from HEK293 cells using the Nuclei Isolation Kit Nuclei EZ Prep (NUC101, Sigma). 5 µl of supernatants from DNase1 mutants expressing HEK293 cells, murine serum, urine, or bile (the latter 1:10 diluted in assay buffer) were mixed with chromatin purified from 1.5 x 10^6^ HEK293 cells in 20 mM Tris-HCl pH 7.4, 2 mM MgCl_2_, 2 mM CaCl_2_, 50 mM NaCl with 1x HALT protease inhibitor (78425, Thermo Scientific). The mixture was incubated at 37°C for 1 h and then heated for an additional 10 min at 65°C. The QIAmp DNA Blood Mini Kit (51106, Qiagen) was used to isolate digested chromatin DNA from the mixture. Eluted material was separated in a 2% agarose gel at 120 V for 45 min. DNA fluorescence of the SYBR Safe stained gels was recorded with the Bio-Rad ChemiDoc Imager. ImageJ was used to quantify fluorescence intensity of digested high molecular chromatin aggregates on a grayscale inverted LUT image. To overcome inter-animal variability of transgenic mice and low signal intensity, we scanned the entire DNA signal including high molecular weight chromatin aggregates and the DNA-fragment ladder pattern up to the last visible signal in chromatin degradation gels shown in **Figure 4**.

### 4.3 Neutrophil isolation

Neutrophils were isolated from citrate anticoagulated blood (02.1067.001, Sarstedt) as previously described (44). In brief, blood was layered onto Histopaque 1119 (11191, Sigma Aldrich). After centrifugation for 20 min at 800 × g, the neutrophil-rich layer was collected. The cells were washed with Hanks-buffered salt solution without divalent cations (HBSS; 14170112, Life Technologies) supplemented with 5 mM EDTA (E3889, Sigma Aldrich) and 0.1% bovine serum albumin (BSA; A1470, Sigma-Aldrich). Washed cells were fractionated on a discontinuous Percoll gradient (17544501, GE Healthcare). After centrifugation for 20 min at 800 × g, the neutrophil-rich layer was collected and washed with 0.1% BSA in HBSS-. All procedures were conducted at room temperature. Neutrophil viability was > 98%, as determined by trypan blue (15250-061, Sigma Aldrich) exclusion.

### 4.4 *In vitro* NET degradation

Purified neutrophils (1 x 10^6^ per well) were seeded in serum-free Dulbecco’s Modified Eagle’s Medium (DMEM; 41965039, Life Technologies) onto 96-well microplates. Neutrophils were activated with 100 nM phorbol 12-myristate 13-acetate (PMA; P8139, Sigma Aldrich) for 4 h at 37°C. 10-fold diluted supernatants of DNase1 mutants-transfected HEK293 cells (see below) were added and incubated with formed NETs for up to 90 min at 37°C. Plates were washed and DNA was fixed with 2% formalin (P087.1, ROTI Histofix, Roth) in HBSS. DNA was stained with 1 µM of cell-impermeable DNA dye, Sytox Green (S7020, Life Technologies) and fluorescence was recorded (Tecan Spark 10 M). Immunofluorescence images were taken on a Nikon Eclipse Ts2R equipped with the Nikon NIS-Elements software.

### 4.5 Cell transfection

HEK293 cells were cultured in DMEM (41965039, Gibco) supplemented with 10% fetal bovine serum (FBS; 16250078, Life Technologies) and 1% penicillin-streptomycin (15140122, Life Technologies) in T75 tissue culture flasks (83.3911.002, Sarstedt) at 37°C in a humified atmosphere and 5% CO_2_. 60% confluent cells were transiently transfected with DNases coding pcDNA3.1 vectors using polyethyleneimine (PEI; 23966-2, Polysciences). After 48h transfection supernatants were removed and centrifuged for 5 min at 500 x g.

### 4.6 Protein purification of DNase1 and DNase1L3

DNase1 was produced in stably transfected Chinese hamster ovary (CHO) cells by Genscript. DNase1 was purified from filtered cell supernatant using a ConA 4B HiTrap column (GE Life Science) on an ÄKTA Prime. Purified DNase1L3 was provided by Neutrolis, Inc. (Cambridge, MA, USA).

### 4.7 Preparation of *in vitro* expression vectors

*De novo* synthesized DNase1 variants as part of pLIVE vectors were produced by Genscript. Genes of interest were cloned in pcDNA3.1 vectors using the NheI-HF and XhoI restriction sites. Sequences of all the plasmids were confirmed by DNA sequencing by Seqlab. For amplification, plasmids were transformed in NEB 5-alpha F´I^q^ competent *E.coli* (high efficiency, C2992H, New England Biolabs) and isolated from bacterial cells using PureLink HiPure Plasmid Maxiprep Kit (K210007, Thermo Scientific).

### 4.8 Mice

All mice were bred to a C57BL/6 genetic background for >10 generations. We crossed the previously described mouse lines *Dnase1^-/-^* and *Dnase1l3^-/-^* (14, 65) to generate double-deficient *Dnase1^-/-^Dnase1l3^-/-^* mice.

### 4.9 Production of DNase1^G,K,O^ transgenic mice

We produced *Dnase1^-/-^Dnase1l3^-/-^* mice that express DNase1^G,K,O^ by hydrodynamic tail vein injection as described previously (66, 67). In brief, 50 μg of pLIVE plasmid was diluted in 0.9% saline in a volume equivalent to 10% of the body mass of the mouse. Mice were anaesthetized with isoflurane and the plasmid solution was then injected intravenously over 5 - 8 seconds *via* the tail vein. After 7 days, mice were sacrificed and blood, bile, and urine were collected. Bile was not tested undiluted due to the small volume that can be aspirated from a murine gall bladder.

### 4.10 Statistics

Data are shown as mean ± SEM. Statistical analyses of the data included one sample t-test, one-way and two-way ANOVA followed by Dunnett’s multiple comparisons test, or Kruskal-Wallis followed by Dunn’s test depending on data distribution. We confirmed normal distribution of data using the Shapiro-Wilk test (*p* < 0.05) for each experiment. Results were considered significant at p < 0.05. All statistical analyses were performed with GraphPad Prism version 8.2.0 (GraphPad, USA).

## Data availability statement

The original contributions presented in the study are included in the article/**Supplementary Material**, further inquiries can be directed to the corresponding author.

## Ethics statement

Use of healthy human blood samples for isolation of neutrophils was approved by the “Ärztekammer” Hamburg (#2322). Written informed consent for participation was not required for this study in accordance with the national legislation and the institutional requirements. The animal study was reviewed and approved by 143/15, approved by the Ministry for Health and Consumer Protection in Hamburg, Germany.

## Author contributions

HE, JG, DK, and MO performed experiments. NW, SK, MF, RKM, MB, JCG, RJSP, LMB, JO, CM and EXS conceived and discussed data. ES edited the draft. HE and TAF generated figures. TAF and TR conceived, supervised, and directed the study and provided funding. HE and TR wrote the manuscript. All authors contributed to the article and approved the submitted version.
