# Supplementary material for: Targeting NETs using dual-active DNase1 variants
Source: Front Immunol. 2023 May 23;14:1181761. doi: 10.3389/fimmu.2023.1181761 (PMC10242134; doi:10.3389/fimmu.2023.1181761)
Supplement: Supplementary file 1 [file DataSheet_1.docx]

Supplementary Material

Targeting NETs using dual-active DNase1 variants

Hanna Englert^1^, Josephine Göbel^1^, Danika Khong^1^, Maryam Omidi^1^, Nina Wolska^1^, Sandra Konrath^1^, Maike Frye^1^, Reiner K. Mailer^1^, Manu Beerens^1^, Julian C. Gerwers^1^, Roger J. S. Preston^2^, Jacob Odeberg^3,4^, Lynn M. Butler^1,3,4,5^, Coen Maas^6^, Evi X. Stavrou^7, 8^, Tobias A. Fuchs^1,9^, Thomas Renné^1,2,10*^

*** Correspondence:**Thomas Renné, M.D. Ph.D.
[thomas@renne.net](mailto:thomas@renne.net)


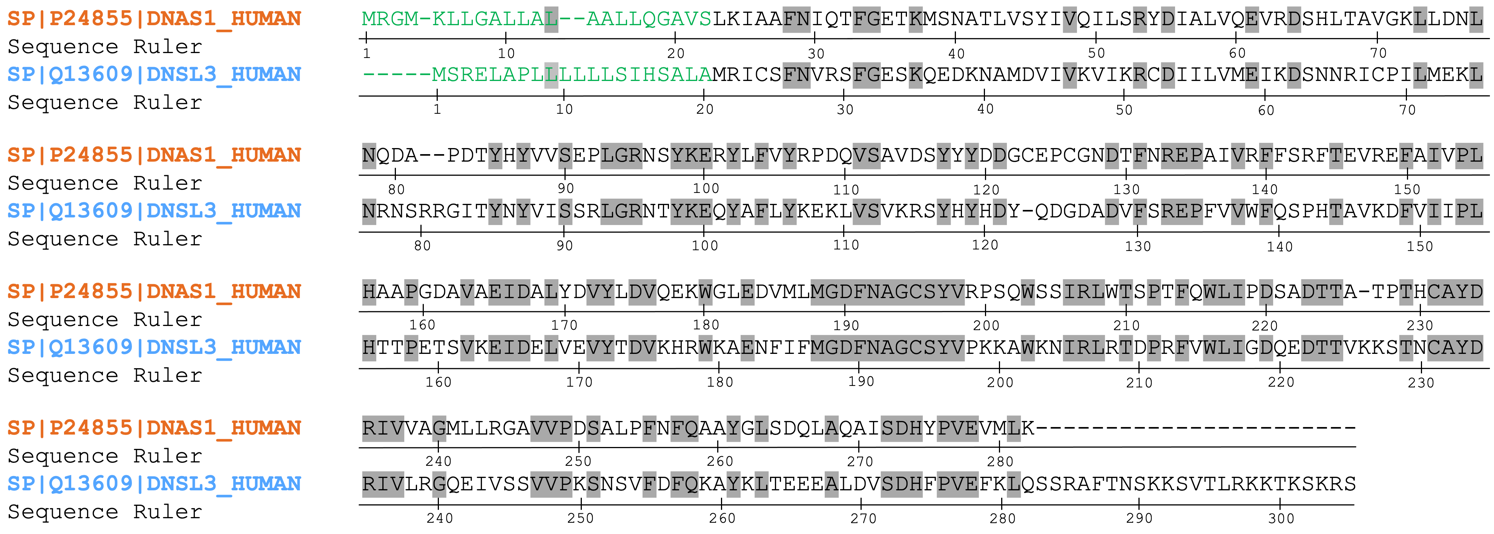


**Supplementary Fig. 1. Alignment of human DNase1 and DNase1L3.**Protein sequence alignment of human DNase1 (SEQ ID P24855, orange) and human DNase1L3 (SEQ ID Q13609, blue), retrieved from the human protein database from Uniprot. The signal peptide is highlighted in green and conserved AAs are depicted in grey.

**Supplementary Table 1. The insertion of non-conserved DNase1L3 amino acids into DNase1 yielded 20 novel enzymatic variants.** Non-conserved amino acid stretches of human DNase1 and DNase1L3 flanked by conserved amino acids of the respective enzyme (N- and C-terminal anchors) are listed. Mutations that transfer sequences from DNase1L3 to DNase1 are shown and categorized into 20 different variants (first column from the right; variants A – T). CA = C-terminal anchor; NA = N-terminal anchor.

| **AA stret-ches** | **N-terminal anchor (NA) in DNase1** | **C-terminal anchor (CA) in DNase1** | **Non-conserved AA DNase1** | **Non-conserved AA DNase1L3** | **DNase1 variants** |
| --- | --- | --- | --- | --- | --- |
| 0 | N/A | N/A | 1-22 (MRGMKLLGALLALAALLQGAVS) | 1-20 (MSRELAPLLLLLLSIHSALA) | A |
| 1 | SP | F28/N29 | 23-27 (LKIAA) | 21-25 (MRICS) | B |
| 2 | F28/N29 | F33/G34 | 30-32 (IQT) | 28-30 (VRS) |  |
| 3 | F33/G34 | K37 | 35-36 (ET) | 33-34 (ES) |  |
| 4 | K37 | V48 | 38-47 (MSNATLVSYI) | 36-45 (QEDKNAMDVI) | C |
| 5 | V48 | R53 | 49-52 (QILS) | 47-50 (KVIK) | D |
| 6 | R53 | D55 | 54 (Y) | 52 (C) |  |
| 7 | D55 | E61 | 56-60 (IALVQ) | 54-58 (IILVM) |  |
| 8 | E61 | D64 | 62-63 (VR) | 60-61 (IK) | E |
| 9 | D64 | L73 | 65-72 (SHLTAVGK) | 63-70 (SNNRICPI) |  |
| 10 | L73 | L77/N78 | 74-76 (LDN) | 72-74 (MEK) |  |
| 11 | L77/N78 | Y85 | 79-84 (QDAPDY) | 77-84 (RNSRRGIT) | F |
| 12 | Y85 | Y87 | 86 (H) | 86 (N) | G |
| 13 | Y87 | S90 | 88-89 (VV) | 88-89 (VI) |  |
| 14 | S90 | L93/G94/R95 | 91-92 (EP) | 91-92 (SR) |  |
| 15 | L93/G94/R95 | Y98/K99/E100 | 96-97 (NS) | 96-97 (NT) | H |
| 16 | Y98/K99/E100 | Y102 | 101 (R) | 101 (Q) |  |
| 17 | Y102 | F104 | 103 (L) | 103 (A) |  |
| 18 | F104 | Y106 | 105 (V) | 105 (L) |  |
| 19 | Y106 | Y111/S112 | 107-110 (RPDQ) | 107-110 (KEKL) | I |
| 20 | Y111/S112 | Y117 | 113-116 (AVDS) | 113-116 (VKRS) |  |
| 21 | Y117 | Y119 | 118 (Y) | 118 (H) |  |
| 22 | Y119 | D121 | 120 (D) | 120 (H) |  |
| 23 | D121 | D129 | 122-128 (GCEPCGN) | 122-127 (YQDGDA) | J |
| 24 | D129 | F131 | 130 (T) | 129 (V) |  |
| 25 | F131 | R133/E134/P135 | 132 (N) | 131 (S) |  |
| 26 | R133/E134/P135 | V138 | 136-137 (AI) | 135-136 (FV) | K |
| 27 | V138 | F140 | 139 (R) | 138 (W) | L |
| 28 | F140 | T145 | 141-144 (FSRF) | 140-143 (QSPH) |  |
| 29 | T145 | F150 | 146-149 (EVRE) | 145-148 (AVKD) |  |
| 30 | F150 | I152 | 151 (A) | 150 (V) |  |
| 31 | I152 | P154/L155/H156 | 153 (V) | 152 (I) |  |
| 32 | P154/L155/H156 | P159 | 157-158 (AA) | 156-157 (TT) |  |
| 33 | P159 | V163 | 160-162 (GDA) | 159-161 (ETS) |  |
| 34 | V163 | E165/I166/D167 | 164 (A) | 163 (K) |  |
| 35 | E165/I166/D167 | L169 | 168 (A) | 167 (E) | M |
| 36 | L169 | V172/Y173 | 170-171 (YD) | 169-170 (VE) |  |
| 37 | V172/Y173 | D175/V176 | 174 (L) | 173 (T) |  |
| 38 | D175/V176 | W180 | 177-179 (QEK) | 176-178 (KHR) | N |
| 39 | W180 | E183 | 181-182 (GL) | 180-181 (KA) |  |
| 40 | E183 | M187-GDFNAGCSY-V198 | 184-187 (DVML) | 183-187 (NFIF) |  |
| 41 | M187-GDFNAGCSY-V198 | W203 | 199-202 (RPSQ) | 198-201 (PKKA) | O |
| 42 | W203 | I206/R207/L208 | 204-205 (SS) | 203-204 (KN) |  |
| 43 | I206/R207/L208 | T210 | 209 (W) | 208 (R) |  |
| 44 | T210 | P212 | 211 (S) | 210 (D) |  |
| 45 | P212 | F214 | 213 (T) | 212 (R) | P |
| 46 | F214 | W216/L217/I218 | 215 (Q) | 214 (V) |  |
| 47 | W216/L217/I218 | D220 | 219 (P) | 218 (G) |  |
| 48 | D220 | D223/T224/T225 | 221-222 (SA) | 220-221 (QE) |  |
| 49 | D223/T224/T225 | T229 | 226-228 (ATP) | 225-228 (VKKS) | Q |
| 50 | T229 | C231-AYDRI-V237 | 230 (H) | 230 (N) | R |
| 51 | C231-AYDRI-V237 | G240 | 238-239 (VA) | 238-239 (LR) |  |
| 52 | G240 | V247/V248/P249 | 241-246 (MLLRGA) | 241-246 (QEIVSS) |  |
| 53 | V247/V248/P249 | S251 | 250 (D) | 250 (K) |  |
| 54 | S251 | F255 | 252-254 (ALP) | 252-254 (NSV) |  |
| 55 | F255 | F257/Q258 | 256 (N) | 256 (D) | S |
| 56 | F257/Q258 | Y261 | 259-260 (AA) | 259-260 (KA) |  |
| 57 | Y261 | L263 | 262 (G) | 262 (K) |  |
| 58 | L263 | A268 | 264-267 (SDQL) | 264-267 (TEEE) |  |
| 59 | A268 | S272/D273/H274 | 269-271 (QAI) | 269-271 (LDV) |  |
| 60 | S272/D273/H274 | P276/V277/E278 | 275 (Y) | 275 (F) | T |
| 61 | P276/V277/E278 | L281 | 279-280 (VM) | 279-280 (FK) |  |
| 62 | L281 | - | 282 (K) | 282-305 (QSSRAFTNSKKSVTLRKKTKSKRS) |  |

**Supplementary Table 2. Statistical comparison of novel enzymatic variants with native** **DNase1 and DNase1L3.** We analyzed statistical differences in DNase1 (SRED, Fig. 2B) and DNase1L3 (chromatin degradation assay, Fig. 2C) activities of the variants compared to native DNase1 and DNase1L3. One sample t-tests were performed. p < 0.05 is given in bold.

| **DNase1 variants** | **Significance level (*vs*. DNase1)** | **Significance level (*vs*. DNase1L3)** |
| --- | --- | --- |
| A | 0.7706 | 0.1982 |
| B | **0.0442** | 0.0852 |
| C | 0.0506 | 0.1178 |
| D | **0.0487** | 0.4968 |
| E | **0.0303** | 0.5681 |
| F | 0.1359 | 0.3000 |
| G | 0.1898 | 0.7299 |
| H | 0.0513 | **0.0394** |
| I | **0.0339** | **0.0053** |
| J | 0.6684 | 0.4864 |
| K | **0.0255** | 0.5471 |
| L | **0.0413** | 0.1400 |
| M | 0.5394 | **0.0352** |
| N | 0.3377 | **0.0005** |
| O | **0.0006** | 0.7142 |
| P | 0.1345 | **0.0088** |
| Q | 0.3061 | 0.3020 |
| R | 0.6667 | **0.0055** |
| S | 0.2419 | **0.0401** |
| T | **0.0365** | 0.2601 |
